# Role of mitochondria in inflammatory lung diseases

**DOI:** 10.3389/fphar.2024.1433961

**Published:** 2024-08-20

**Authors:** Venkata Ramireddy Narala, Sahithi Reddy Narala, Parasuraman Aiya Subramani, Kalpana Panati, Narasaiah Kolliputi

**Affiliations:** ^1^ Department of Zoology, Yogi Vemana University, Kadapa, India; ^2^ Kurnool Medical College, Kurnool, India; ^3^ Thünen Institute of Fisheries Ecology, Bremerhaven, Germany; ^4^ Department of Biotechnology, Government College for Men, Kadapa, India; ^5^ Division of Allergy and Immunology, Department of Internal Medicine, Morsani College of Medicine, University of South Florida, Tampa, FL, United States

**Keywords:** inflammation, lung diseases, mitochondrial dysfunction, oxidative stress, therapeutic targets

## Abstract

Mitochondria play a significant and varied role in inflammatory lung disorders. Mitochondria, known as the powerhouse of the cell because of their role in producing energy, are now recognized as crucial regulators of inflammation and immunological responses. Asthma, chronic obstructive pulmonary disease, and acute respiratory distress syndrome are characterized by complex interactions between immune cells, inflammatory substances, and tissue damage. Dysfunctional mitochondria can increase the generation of reactive oxygen species (ROS), triggering inflammatory pathways. Moreover, mitochondrial failure impacts cellular signaling, which in turn affects the expression of molecules that promote inflammation. In addition, mitochondria have a crucial role in controlling the behavior of immune cells, such as their activation and differentiation, which is essential in the development of inflammatory lung diseases. Their dynamic behavior, encompassing fusion, fission, and mitophagy, also impacts cellular responses to inflammation and oxidative stress. Gaining a comprehensive understanding of the intricate correlation between mitochondria and lung inflammation is essential in order to develop accurate treatment strategies. Targeting ROS generation, dynamics, and mitochondrial function may offer novel approaches to treating inflammatory lung diseases while minimizing tissue damage. Additional investigation into the precise contributions of mitochondria to lung inflammation will provide significant knowledge regarding disease mechanisms and potential therapeutic approaches. This review will focus on how mitochondria in the lung regulate these processes and their involvement in acute and chronic lung diseases.

## 1 Introduction

More fundamental than asthma, yet with limited understanding, mitochondrial defects or dysfunction may play a pivotal role in driving inflammation and/or injury, potentially serving as a common mechanism in the progression of inflammatory lung diseases (Zhou et al., 2021). Mitochondria, versatile organelles involved in cellular energy production, apoptosis regulation, and the orchestration of various biosynthetic pathways, have emerged as key players in the function and viability of leukocytes, central to cytokine production and regulation (Picca et al., 2021; Casanova et al., 2023). Moreover, both immunological and environmental stressors implicated in lung disease etiology directly impact mitochondrial function (Aghapour et al., 2020). Despite supporting evidence, there is a paucity of literature proposing mitochondrial processes as a mechanism in lung diseases, resulting in their underrepresentation in disease-specific research. Inflammatory lung diseases pose an ongoing and escalating public health challenge globally, encompassing conditions such as chronic obstructive pulmonary disease (COPD), asthma, acute lung injury (ALI), acute respiratory distress syndrome (ARDS), and cystic fibrosis, burdening healthcare systems with significant morbidity and mortality (Viegi et al., 2020). COPD, in particular, is projected to rank as the third leading cause of death and the fifth leading cause of disability-adjusted life years lost worldwide by 2020 (Li H. et al., 2022a). Despite extensive research efforts, the precise mechanisms underlying chronic and acute lung inflammation remain incompletely elucidated. However, it is evident that active inflammation and/or injury disrupt cellular machinery, leading to functional and survival deficits.

### 1.1 Overview of inflammatory lung diseases

Lung disease is a broad term that encompasses various types of diseases affecting the lungs. These include ALI, ARDS, COPD, asthma and cystic fibrosis (Panati et al., 2020). Asthma is a chronic inflammatory airway disease characterized by reversible airflow obstruction, bronchial hyperresponsiveness, and underlying inflammation. It affects millions worldwide and manifests through symptoms such as wheezing, shortness of breath, chest tightness, and coughing (Shaik et al., 2015). The pathogenesis of asthma involves a complex interplay between genetic predisposition and environmental factors. Exposure to allergens, pollutants, respiratory infections, and stress can trigger immune responses, leading to airway inflammation. The hallmark of asthma pathophysiology is the infiltration of airway walls by inflammatory cells, such as eosinophils, T-helper 2 (Th2) lymphocytes, and mast cells (Shaik et al., 2015). This results in the release of cytokines and chemokines that perpetuate inflammation and contribute to structural changes in the airway, known as remodeling.

Treatment of asthma focuses on controlling symptoms and preventing exacerbations but there is no cure. Pharmacological management includes the use of inhaled corticosteroids (ICS) as the cornerstone of therapy, which reduces inflammation. Long-acting beta-agonists (LABAs) are often used in combination with ICS to improve bronchodilation. For severe cases, biologics targeting specific inflammatory pathways, such as anti-IgE, anti-IL-5, or anti-IL-4/13, have shown efficacy (Sarkar et al., 2024). Emerging research highlights the significant role of mitochondria in inflammatory lung diseases, including asthma will be discussed in the following sections.

Inflammation in the lungs and airway cells of patients with lung disease, as well as in animal models of lung injury, has been linked to structural and functional changes in mitochondria (Narala et al., 2018; Cloonan et al., 2020). Mitochondrial bioenergetics, the production of energy within mitochondria, is found to be decreased in inflammatory lung diseases (Riou et al., 2020). This decrease is associated with increased expression of uncoupling proteins and a loss of inner membrane potential, leading to impaired ATP synthesis. As a result, the mitochondria become more susceptible to a process called the mitochondrial permeability transition pore (MPTP) due to oxidative stress and lack of ATP. This can ultimately result in cell death, either through apoptosis or necrosis, depending on the severity of mitochondrial dysfunction and ATP loss (Cloonan et al., 2020; Supinski et al., 2020; Zhou et al., 2021). The increased apoptosis of alveolar epithelial cells and fibroblasts, two types of cells in the lungs, can contribute to the impaired healing of wounds and maintenance of the normal alveolar epithelial barrier (Conforti et al., 2020; John et al., 2021). This is particularly important in lung injury, ARDS, and idiopathic pulmonary fibrosis, as the damage and failure to repair the epithelial cells play a major role in the development of these diseases. Fibroblasts and myofibroblasts are also essential in maintaining the normal structure of the lungs and promoting healing after injury. However, they can also contribute to fibrotic changes in the lungs. Mitochondrial dysfunction, along with the loss of cytochrome c and alterations in cellular redox status, has been shown to affect transforming growth factor beta (TGF-β) signaling in fibroblasts, promoting the development of fibrosis (Bueno et al., 2020; Mohanan et al., 2024). Additionally, mitochondrial reactive oxygen species (ROS) can directly stimulate changes in alveolar epithelial cells, causing them to adopt a profibrotic phenotype (Larson-Casey et al., 2020; Selman and Pardo, 2020). Structural and functional alterations of mitochondria have been identified in both the inflamed lungs and airway cells of patients with lung disease and in animal models of lung injury (Cloonan et al., 2020; Sharma et al., 2021). Mitochondrial bioenergetics has been found to be depressed in inflammatory lung diseases, associated with increased expression of uncoupling proteins and loss of inner membrane potential with impaired ATP synthesis. This is believed to lead to activation of the MPTP due to oxidative stress and depletion of ATP, resulting in apoptosis or necrosis, dependent on the severity of mitochondrial dysfunction and the degree of ATP loss (Visentin et al., 2020; Danieli et al., 2023). Increased apoptosis of alveolar epithelial cells and fibroblasts can contribute to impairment of wound repair and maintenance of the normal alveolar epithelial barrier, which has particular importance in lung injury, ARDS, and idiopathic pulmonary fibrosis where epithelial injury and disrepair is a major pathogenic factor. Fibroblasts and myofibroblasts are also vital in maintaining normal lung structure and repair following injury, but their differentiation and function can contribute to fibrotic changes. Mitochondrial dysfunction with loss of cytochrome c and changes in cellular redox status has been shown to alter TGF-β signaling in fibroblasts, promoting fibrogenesis, and mitochondrial ROS can directly stimulate alveolar epithelial cell changes towards a profibrotic phenotype (Henderson and O’Reilly, 2021; Siekacz et al., 2021). Our earlier studies suggest that IL-6 induces Bcl-2 expression to perform cytoprotective functions in response to oxygen toxicity, and that this effect is mediated by alterations in the interactions between Bak and mitofusin (MFN) proteins (Kolliputi and Waxman, 2009; Waxman and Kolliputi, 2009). Mitochondrial A-kinase anchoring protein 1 (Akap1) has been shown to regulate mitochondrial function in hyperoxic lung injury (Narala et al., 2018; Sidramagowda Patil et al., 2022; Soundararajan et al., 2022). The aforementioned studies clearly indicate the significant contribution of mitochondria in inflammatory lung diseases.

### 1.2 Importance of understanding mitochondrial involvement

A heightened understanding of the basic mechanisms underlying inflammatory lung disease is likely to reveal key opportunities for successful intervention. To date, the study of lung inflammation has focused primarily on the actions of pro-inflammatory mediators and cells. However, recent reports revealing the importance of homeostatic cellular functions in the regulation of immune responses have uncovered an exciting new area for investigation. In particular, the intracellular organelle responsible for generation of most of the cellular energy, the mitochondrion, has emerged as a central regulator of immune responses and has been shown to contribute to the pathogenesis of numerous immune and inflammatory mediated diseases. Information is now accumulating regarding the importance of mitochondrial regulation of energy dependent cellular functions in immune and epithelial cells during host responses to infection and in the regulation of lymphocyte activation within the lung (Riou et al., 2020). These are key processes for the clearance of pathogens but when excessive can lead to immune mediated lung injury and the development of chronic inflammatory lung diseases. Regulation of these processes by mitochondria in the lung and their contribution to acute and chronic lung diseases will be covered in the subsequent review.

## 2 Mitochondrial dysfunction in inflammatory lung diseases

**FIGURE 1:**
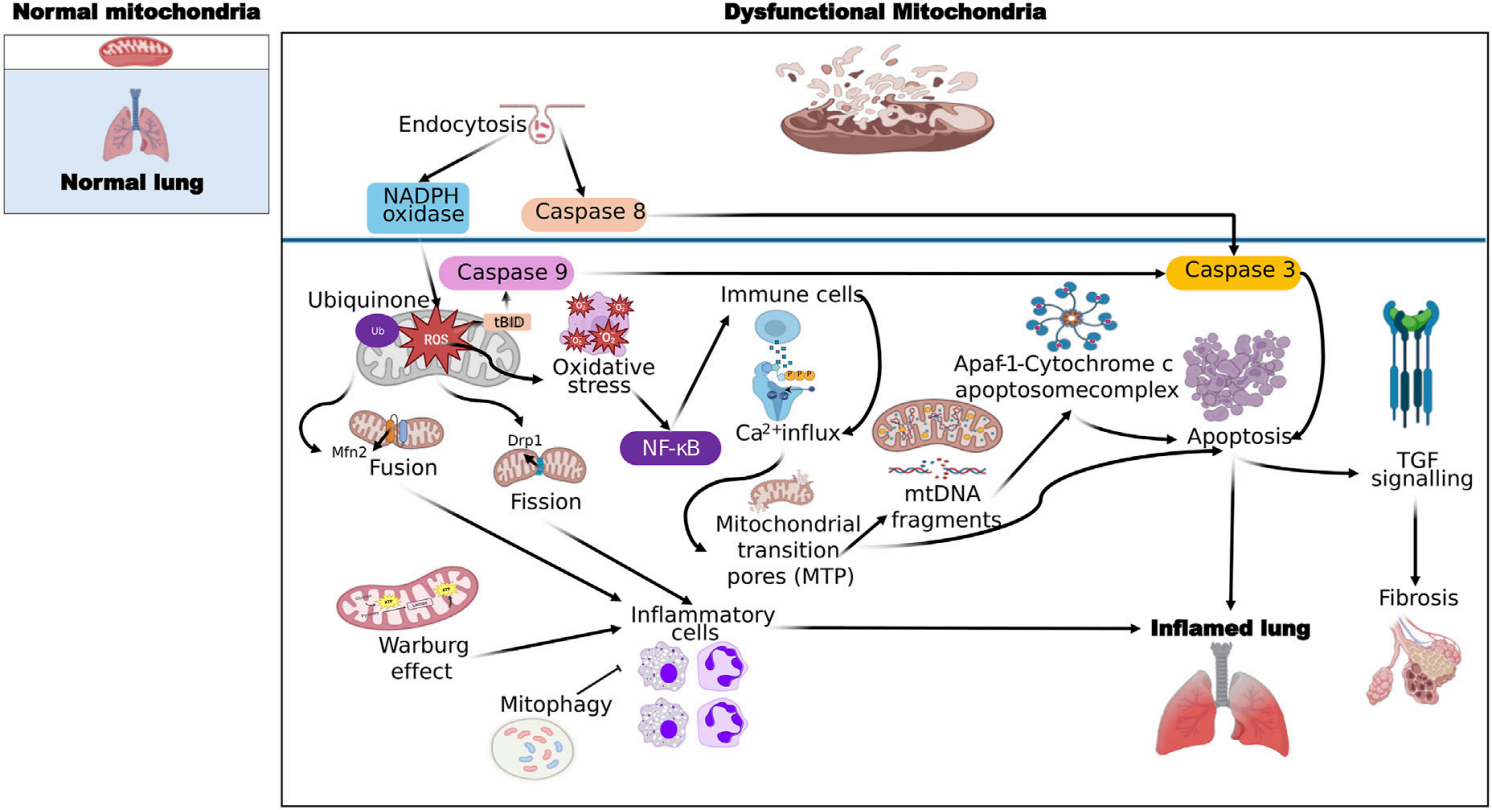
General mechanisms of ROS production by mitochondrial dysfunction leading to lung inflammation and fibrosis. Molecules that activate the next step are connected by sharp arrows (→), and inhibition is indicated by blunt arrows (┴), please refer to the text for more details.

In inflammatory lung diseases, mitochondrial dysfunction is a precursor to apoptosis and necrosis. Mitochondria are the energy producing organelles and are involved in the regulation of cellular processes and signaling pathways. Mitochondrial-driven apoptosis can occur through the intrinsic (mitochondrial) or extrinsic (cell death receptor) pathways, although the extrinsic pathway requires the intrinsic pathway for amplification of the death signal (Martini and Passos, 2023). The intrinsic pathway is initiated by various stimuli that increase the permeability of the mitochondrial outer membrane, allowing the release of apoptogenic factors from the intermembrane space. The proteins released from the intermembrane space include cytochrome c, which is required for the formation of a complex with dATP and apoptotic protease activating factor 1, known as the apoptosome (Morse et al., 2024; Zhou et al., 2024). This complex activates procaspase 9 and the downstream caspases that are involved in the execution of cell death. Cytochrome c release and subsequent caspase activation are also associated with a caspase-independent form of cell death termed necrosis (Figure 1). Mitochondrial damage associated with apoptosis and necrosis can release damage-associated molecular pattern molecules (DAMPs) and histones that activate inflammatory pathways to exacerbate the lung disease (Koenig and Buskiewicz-Koenig, 2022).

### 2.1 Mechanisms of mitochondrial dysfunction

Mitochondrial dysfunction can arise through a wide array of genetic and environmental factors and has been implicated in a wide variety of pathological states. ATP is produced via oxidative phosphorylation, a process which occurs within the inner mitochondrial membrane and involves the movement of electrons between complexes (I-IV) which in turn pump protons across the inner mitochondrial membrane (Boyman et al., 2020; Reiter et al., 2021). ATP synthase then uses the proton motive force to generate ATP from ADP. Any interruption to this process or uncoupling of the electron transport chain can decrease ATP production and increase electron leakage. This can occur under conditions of inflammation and subsequently decrease ATP levels. Pro-inflammatory mediators such as cytokines induce the expression of inducible nitric oxide synthase (iNOS) and increased NO production can combine with ROS to form peroxynitrate, which is a potent inhibitor of mitochondrial respiration and energy production (Nesci et al., 2021). In addition to decreased ATP production, mitochondria can also release apoptotic factors under the influence of ROS such as cytochrome c and apoptosis-inducing factor. Cytochrome c can activate the caspase cascade resulting in cellular apoptosis and loss of function (Kim et al., 2020). These disruptions to cellular energy metabolism and induction of apoptosis can have severe consequences for lung cells and exacerbate underlying lung disease.

### 2.2 Impact of mitochondrial dysfunction on inflammation

Inflammatory response occurs when lung tissue is injured. It is a complicated and highly organized process, which can restore the structure and function of the tissue and clear away injurious stimuli. The inflammatory response to infection has been shown to cause changes in energy requirements of the cells. When everything goes well in an inflammatory response, mitochondria are central to many of the cell energy-dependent events including changes in cell permeability and phagocytosis as well as providing secondary messengers for the many signaling pathways which regulate the inflammatory response (López-Armada et al., 2013). However, when inflammation is excessive or chronic, as in many diseases, the consequences for mitochondria can be dire. Excess reactive oxygen/nitrogen species (ROS/RNS) and changes in intracellular Ca^2+^ levels can lead to what is termed MPTP formation (Rottenberg and Hoek, 2017). MPTP formation has been linked with cell apoptosis and necrosis as well as release of cytochrome c from the inner membrane space into the cytosol which can induce further caspase dependent cell death (Crompton, 1999; Murphy, 1999). Both cell death and decrease in membrane potential can lead to a lack of ATP production (Vorobjeva et al., 2020; Bernardi et al., 2023; Flores-Romero et al., 2023).

#### 2.2.1 Role of dysfunctional mitochondria in the production of different classes of cytokines involved in pulmonary inflammation

**FIGURE 2:**
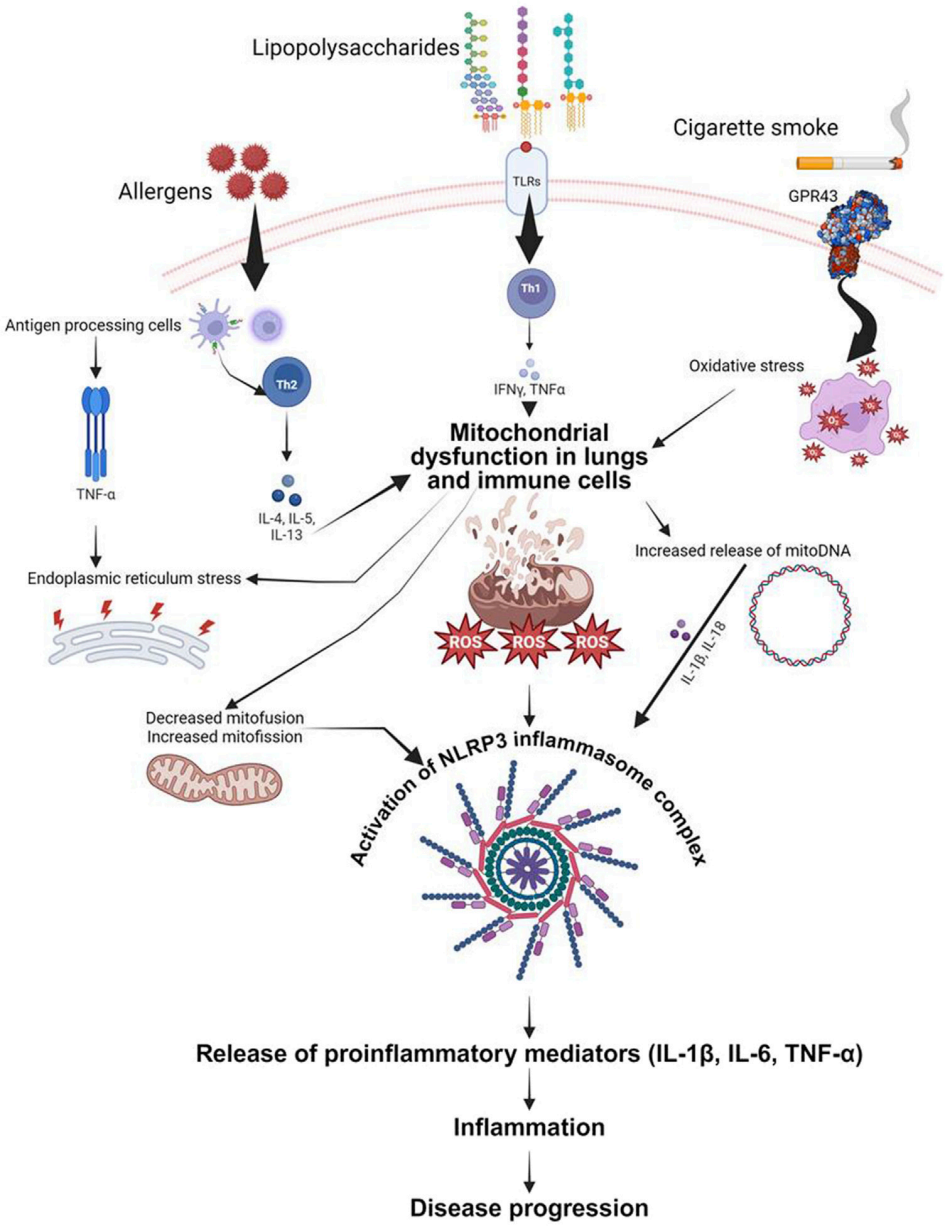
Schematic representation of proposed mechanisms by which mitochondrial dysfunction contributes to airway inflammation. Both airway epithelial cells and immune cells are depicted as sources and targets of mitochondrial-derived reactive oxygen species (ROS). These ROS, induced by mitochondrial defects, trigger the release of pro-inflammatory cytokines (IL-6, IL-1β, TNF-α) in response to various stimuli including allergens, bacterial lipopolysaccharide, and cigarette smoke. The figure highlights the role of mitochondrial DNA (mitoDNA) damage and NLRP3 inflammasome activation in exacerbating inflammatory responses. Key signaling pathways and cellular processes involved in the mitochondrial regulation of inflammation are illustrated, emphasizing potential therapeutic targets for mitigating airway inflammation in asthma, acute lung injury, and chronic obstructive pulmonary disease.

Recent research has brought attention to the important role of mitochondria in the generation of cytokines that contribute to inflammation in diseases like asthma and other chronic lung conditions. One of the key pathways involved is the activation of the NLRP3 inflammasome by mitochondrial reactive oxygen species (mtROS). In conditions such as asthma and COPD, mitochondrial dysfunction leads to increased mtROS production, which activates the NLRP3 inflammasome. This activation triggers the release of pro-inflammatory cytokines like IL-1β, contributing to inflammation and disease progression (Chen X. et al., 2023a) (Figure 2). Fragmented oxidized mtDNA enters the cytosol where it activates NLRP3 inflammasome and generates the production of IL-1β, IL-18, and cyclic GMP-AMP synthase-STING, inducing type I interferons and interferon-stimulated genes (Orekhov et al., 2023).

In asthma, mitochondrial dysfunction is linked to Th2-driven inflammation characterized by cytokines such as IL-4, IL-5, and IL-13. Mitochondrial-targeted antioxidants have shown promise in reducing oxidative stress and inflammatory responses in asthma models (Caldeira et al., 2021). Furthermore, studies on idiopathic pulmonary fibrosis (IPF) have demonstrated that increased mtROS promotes fibrogenesis through TGF-β signaling. Therapeutic strategies targeting mitochondrial oxidative stress and mitochondrial dynamics, such as using mitochondrial antioxidants and fission inhibitors, have shown potential in controlling fibrosis and inflammation in IPF (Caldeira et al., 2021).

Overall, these findings underscore the significant impact of mitochondrial function and dysfunction on cytokine production and inflammation in chronic lung diseases, paving the way for novel therapeutic approaches targeting mitochondrial pathways (Kamp et al., 2011; Caldeira et al., 2021) (Figure 2).

### 2.3 Role of mitochondria in disease progression

Disease progression is a central theme in human pathology and is the overwhelming determinant in the morbidity and mortality associated with all diseases, especially those associated with chronic inflammation. Although the clinical manifestations and underlying etiologies of specific chronic inflammatory diseases such as COPD, asthma, IPF, and sarcoidosis can vary considerably, the common thread is that in all cases disease progression is associated with respiratory insufficiency and failure (Hatipoğlu and Aboussouan, 2022; Bhat et al., 2023). The factors that drive disease progression are poorly understood, but it is generally considered that a combination of aberrant repair processes and/or repeated acute exacerbations of the disease are involved. This leads to the tissue destructive events that are responsible for the development of respiratory insufficiency. In this context, there is some emerging data to suggest that mitochondria may have an important role in disease progression, especially through the effects of mitochondria on cellular energy production and the induction of apoptosis (Protasoni and Zeviani, 2021). Therefore, the effects of mitochondria on energy balance and apoptosis in specific diseases will be discussed.

Energy balance is a critical determinant of cell fate, and insufficient energy availability is a common feature in different cells and tissues in a variety of chronic inflammatory diseases. Although *in vivo* energy status of specific cells and tissues is difficult to assess, the finding of decreased ATP levels and increased AMP-activated protein kinase activation in peripheral lung tissue from COPD and IPF patients suggests that energy imbalance may be important in diseases of the lung (Ornatowski et al., 2020). Mitochondria are central regulators of energy production and play pivotal roles in the cellular stress response and induction of apoptosis, and thus it is logical to propose that mitochondrial dysfunction may be a causative factor in energy imbalance and a key player in disease progression in various lung diseases.

## 3 Mitochondrial reactive oxygen species in inflammation

Physiologically, ROS target and damage the components of the electron transport chain (ETC.), causing the escape of electrons and reducing molecular oxygen to form superoxide ions (Juan et al., 2021). A small amount of ROS oxidizes and damages intracellular proteins and activated transcription factors as “second messengers” in cell signaling pathways, a process which is essential for cell survival, reproduction, and adaptation to stimuli (Juan et al., 2021). Oxidative stress is defined as an imbalance between pro-oxidants and antioxidants that leads to a disruption of redox signaling and control of cellular function. However, excessive ROS production can lead to a vicious cycle of oxidative stress, creating further damage to ETC components which can propagate more electron leakage and ROS production (Zhao et al., 2019). Redox signaling is now known to be involved in the pathology of inflammation, oxidative stress-induced disease, and cancer. It has long been recognized that infection and inflammation trigger an enhanced production of ROS by a stimulated immune system and by inflamed tissue. This is primarily due to the upregulation of phagocyte NADPH oxidase activity, which is a potent producer of superoxide ions (Vermot et al., 2021). However, production of ROS in inflamed tissue via NADPH oxidase is still surpassed by the same process in mitochondria, and it has been demonstrated that a NADPH oxidase-derived increase in superoxide in the cytosol of inflammation-activated endothelial cells causes an increase in mitochondrial ROS (Figure 1).

### 3.1 Generation and regulation of ROS in mitochondria

Ubiquinone, an essential electron carrier, is thought to regulate mitochondrial ROS creation (Hernansanz-Agustín and Enríquez, 2021). When complex I activity is low, more ubiquinone is in a reduced state, and it donates electrons to O_2_, generating superoxide. Conversely, when complex I activity is high, more of the ubiquinone pool will be in an oxidized state, and this form is incapable of donating electrons. Hence, low complex I activity may lead to high superoxide production in mitochondria. Since many pathological conditions are associated with decreased complex I activity and increased ubiquinone reduction, understanding the mechanisms behind ubiquinone-mediated ROS generation may be important. Superoxide can be generated from other sites in the electron transport chain as well and can be converted to other forms of ROS. Stepwise one-electron reductions of O_2_ yield O_2_^−^, which yields hydrogen peroxide (H_2_O_2_) and finally converts to OH^−^ (Fujii et al., 2022). The latter molecule is especially harmful due to its high reactivity with neighboring biomolecules. Mitochondrial manganese superoxide dismutase (MnSOD) is only capable of scavenging superoxide within the mitochondrial matrix (Candas and Li, 2014). As such, the H_2_O_2_ produced from O_2_^−^ can be detoxified by mitochondrial or cytoplasmic catalase or can yield the highly diffusible H_2_O_2_. An increase in overall mitochondrial ROS generation will have a significant impact on cellular redox balance (Kitada et al., 2020; Latham et al., 2024).

### 3.2 ROS-mediated inflammatory signaling pathways

NF-κB is a key regulator of inflammatory and immune responses and increases the expression of pro-inflammatory cytokines such as TNF-α and IL-1β by up-regulating gene expression through enhanced transcription activating mRNA stabilization (Shen et al., 2019; Zhou et al., 2021). It is likely that NF-κB activation would explain the general increase in pro-inflammatory gene expression caused by oxidative stress and has been shown to be upregulated in a number of acute and chronic inflammatory lung diseases. NF-κB is not only activated by ROS, but its activation can also be prolonged through inhibition of the deactivating enzyme A20. This was shown to be due to increased H_2_O_2_-mediated aryl hydrocarbon receptor nuclear translocation and A20 inhibition, providing a positive feedback loop for increased ROS and pro-inflammatory gene expression (Nakajima and Kitamura, 2013; Jomova et al., 2023) (Figure 2).

Encouraging advances made in the realm of redox biology have unveiled a host of complex inflammatory signaling mechanisms mediated by ROS, which may have profound implications for inflammatory lung diseases. Detailed discussion of these pathways goes beyond the scope of this article, and the following serves as an overview and highlight of recent in-depth reviews on this topic. Broadly speaking, ROS can regulate the activity of redox-sensitive transcription factors and gene expression. ROS are potent activators of the transcription factor NF-κB, and this pathway has been the subject of intense research (Carbonell and Gomes, 2020; Lennicke and Cochemé, 2021; Lind et al., 2024).

### 3.3 Consequences of excessive ROS production

While low and moderately elevated levels of ROS play a role in cellular signaling and maintaining cellular homeostasis, excessive production is associated with cell damage and cell death. In response to oxidative stress, mitochondrial DNA (mtDNA) is highly susceptible to damage due to its proximity to the ETC, lack of protective histones, and inefficient DNA repair mechanisms. Oxidative damage to mtDNA leads to a positive feedback loop in ROS production as the ETC enzymes encoded by mtDNA are damaged, impairing the ETC and increasing electron leakage (Huang et al., 2020; Harada et al., 2023). Moreover, ROS can directly damage the ETC, oxidizing its protein constituents and decreasing its efficiency, thereby increasing electron leakage and ROS generation. These factors contribute to a downward spiral in mitochondrial function and have been implicated in a number of chronic and age-related diseases. Less is known about the specific consequences of mitochondrial ROS in inflammatory lung diseases; however, oxidative damage to mtDNA and the ETC are likely to exacerbate the inflammatory process and contribute to disease chronicity. Conversely, it has been shown that site-directed antioxidants which specifically target mitochondria are effective in inhibiting excessive ROS production and suppressing inflammation. This suggests that increased understanding of the mechanisms by which mitochondrial ROS exacerbates inflammation may lead to the development of more targeted and effective antioxidant therapies for inflammatory lung diseases. Antioxidants targeting first generation ROS have been developed but are largely ineffective in clinical trials (Rice et al., 2011; Li et al., 2015). Consequently, recent research has turned to targeting more stable secondary intermediates, such as 4-HNE. 4-HNE is a reactive aldehyde produced during oxidative stress by lipid peroxidation (Esterbauer et al., 1991; Gomes et al., 2014). Earlier studies show that 4-HNE is elevated in lung disease especially in ALI/ARDS patients (Quinlan et al., 1994; Dalle-Donne et al., 2006). 4-HNE is toxic because it targets 30% of mitochondrial proteins, which results in mitochondrial injury and impaired bioenergetics (Galam et al., 2015).

## 4 Mitochondrial DNA (mtDNA) and inflammation

Mitochondrial genetic material is normally confined in the mitochondria. This enhanced permeability transition is activated in lung cellular stress conditions, such as exposure to excessive ROS, hypoxia, or stressful inflammatory situations involving excessive cytokine production (Bonora et al., 2022). Once the transition pore has been formed in these situations, mtDNA is released into the cytoplasm, although the consequences of mtDNA release from specific lung cellular stress conditions on mtDNA amounts in cytoplasm and subsequent inflammatory responses have yet to be studied in detail (Moya et al., 2021). Evidence on the precise mechanisms involved in the release of mtDNA into the cytoplasm is sparse. However, it is believed that it is due to enhanced permeability transition in the mitochondrial inner membrane, which results in the formation of a transition pore (Lechuga-Vieco et al., 2021; Stewart and Chinnery, 2021).

### 4.1 Release and recognition of mtDNA

The release of mtDNA has been implicated as a potent DAMP that leads to inflammatory responses. Although the precise mechanisms linking this to lung inflammation have not been fully elucidated, the likelihood that this may be involved in lung diseases is suggested by the vast and growing evidence for the role of mtDNA in sterile inflammatory conditions (Riley and Tait, 2020; Zanini et al., 2023). Classical necrosis is distinct from apoptosis in that it leads to cellular swelling, organelle damage and cell lysis with subsequent loss of cellular contents into the extracellular space. Mitochondria are affected by this process with loss of membrane potential and swelling followed by rupture of the outer mitochondrial membrane (OMM) and subsequent release of its contents including cytochrome c and other apoptotic mediators as well as mtDNA. Mitochondrial changes observed in necrotic cell death and the subsequent release of mtDNA have been shown to induce inflammatory responses in liver and cardiac tissues (Pérez-Treviño et al., 2020; Riley and Tait, 2020; Moya et al., 2021). It is possible that this may be similarly implicated in lung inflammation and injury given the now recognized importance of necrosis in regulation cell death pathways and the influence this has in pathological disease.

Apoptosis has been shown to be an important mode of cell death in several lung diseases and more recently it is appreciated that some cell types can undergo caspase-independent apoptosis (Elmore, 2007). This involves permeabilization of the OMM with subsequent release of apoptotic factors into the cytoplasm and final release from the cell as so-called apoptotic bodies (Elmore, 2007). Although the final fate of apoptotic bodies and their contents has not been fully defined, it is possible that mtDNA within could also serve as a chemoattractant or DAMP for inflammatory cells (Faas and de Vos, 2020; Marchi et al., 2023). This mode of cell death is of particular interest regarding the type II alveolar epithelial cell and the broader implications of mtDNA from apoptotic cells and necrotic mtDNA on the inflammatory responses it induces warrants further investigation.

### 4.2 mtDNA-induced inflammatory responses

Akin to bacteria, mtDNA contains hypomethylated CpG motifs that are potent inducers of inflammation (Nakayama and Otsu, 2018). The unmethylated CpG motifs are recognized by toll-like receptor 9 (TLR9), an endosomal receptor that signals through MyD88 to induce NF-κB activation. Triggering of TLR9 by synthetic CpG-DNA has been shown to augment LPS-induced lung inflammation (Knuefermann et al., 2007). A recent study has demonstrated that mtDNA is a powerful stimulant for inflammatory responses in the lung (Long et al., 2022) (Figure 2).

Pulmonary transfection of mtDNA by intratracheal instillation induced profound recruitment of neutrophils and expression of proinflammatory cytokines TNF-α and KC (an IL-8 homologue) in C57BL/6 mice (Long et al., 2022). Similar to the effects seen by overexpression of MnSOD in the mitochondria, mtDNA induced lung inflammation was enhanced in mice with a deleted aconitase-2 (ACO-2) gene (Kim et al., 2015). This is likely due to increased production of O_2_^•−^ by the mitochondria, leading to oxidation and inactivation of the ACO-2 substrate, and hence further accumulation of citrate to promote increased mtDNA synthesis. mDNA-induced inflammatory responses were not dependent upon the host’s innate immune system, as mtDNA instillation in severe combined immune deficiency mice similarly resulted in profound lung inflammation and injury (Hu and Shu, 2020; Riley and Tait, 2020; Newman and Shadel, 2023). It has been found that incident ARDS in trauma and sepsis patients is linked to elevated mtDNA levels in plasma (Faust et al., 2020). This evidence indicates mtDNA has the potential to promote lung inflammation from a wide variety of pathological conditions. Alteration of mtDNA integrity and the nature of specific damage and repair responses will also have significant effects on inflammatory cytokine expression and resolution and are areas of great interest for further research. But further research is needed to fully understand how mtDNA causes inflammation in ALI/ARDS.

### 4.3 Implications of mtDNA in lung diseases

mtDNA is a key player in the development of inflammatory lung diseases. The ability to sequence the entire mitochondrial genome has allowed for the examination of variation in mtDNA sequences in different populations and in patients with various diseases (Taylor and Turnbull, 2005). There are numerous point mutations and rearrangements that have been associated with an array of human diseases including Parkinson’s disease, Alzheimer’s disease, diabetes, tumors (Wallace et al., 1999; Wallace, 2005) and asthma (Polonikov et al., 2009; Zifa et al., 2012). A recent report has indicated that the production and release of mtDNA in response to LPS is linked to RAD50, which in turn triggers ALI/ARDS by activating the STING and NF-κB signaling pathways, leading to increased secretion of proinflammatory cytokines (Zhan et al., 2021). The study found that ALI was less severe in mice with RAD50 deficiency, suggesting that DNA sensors could be vital in identifying potential drug targets for controlling the unregulated inflammatory response in ALI/ARDS (Zhan et al., 2021). Nakahira et al. investigated how mtDNA can act as a DAMP when released into the cytoplasm or extracellular space due to cellular stress or injury. This can trigger immune responses through pathways such as TLR9 and the NLRP3 inflammasome, leading to inflammation (Nakahira et al., 2015). The study showed mechanistically that mtDNA released into the cytosol can bind to NLRP3, triggering its activation and the subsequent production of inflammatory cytokines like IL-1β, contributing to inflammation in lung tissues (Nakahira et al., 2015) (Figure 2). Damaged mtDNA can perpetuate a cycle of mitochondrial dysfunction and further ROS production, contributing to chronic inflammation (Aghapour et al., 2020).

Cloonan and Choi (2016) examined how oxidative stress leads to mitochondrial damage and how this is implicated in COPD. They found that increased ROS production in mitochondria damages mtDNA, leading to mitochondrial dysfunction. This dysfunction results in a feedback loop where more ROS are produced, perpetuating lung inflammation and damage (Cloonan and Choi, 2016). Elevated levels of circulating mtDNA have been observed in COPD patients, correlating with disease severity and exacerbations. This circulating mtDNA acts as a pro-inflammatory signal, exacerbating the inflammatory response in the lungs. This suggests mtDNA as a potential biomarker and a pathogenic factor in COPD (Ware et al., 2024).

In IPF, mitochondrial dysfunction, including reduced mtDNA copy number and impaired oxidative phosphorylation, has been implicated in epithelial cell injury and fibrosis (Bueno et al., 2015; Bueno et al., 2020). The study showed that PINK1 deficiency impairs mitochondrial homeostasis, leading to increased oxidative stress and cellular apoptosis, which promotes the development of fibrosis in lung tissues (Bueno et al., 2015). Similarly, mitochondrial dysfunction and mtDNA damage have been implicated in the pathogenesis of asthma, potentially through enhanced oxidative stress and altered immune responses (Qian et al., 2022). As a result, targeting mitochondrial function and protecting mtDNA from damage are potential therapeutic strategies for inflammatory lung diseases, with ongoing research focused on antioxidants, mitochondrial biogenesis enhancers, and mtDNA repair mechanisms.

## 5 Mitochondrial dynamics and inflammation

Fusion and fission are dynamic processes by which mitochondria undergo shape change and are central to maintaining mitochondrial integrity. Fusion is the process of merging of two mitochondria and is controlled by at least four dynamin-related GTPases. Mutations in the GTPases optic atrophy 1 (OPA1) and Mfn2 are associated with autosomal dominant optic atrophy and Charcot-Marie-Tooth neuropathy type 2A, and suggest a role for abnormal mitochondrial fusion in the pathogenesis of these human diseases (Larrea et al., 2019). In the case of OPA1, this is thought to be due to the inability of mutated OPA1 to restore normal mitochondrial membrane potential and to promote apoptosis of damaged mitochondria (Belenguer and Pellegrini, 2013). Mfn1 and Mfn2 facilitate the merging of the outer mitochondrial membranes and formation of a mitochondrial “docking” complex (Huo et al., 2022). Mitochondrial inner-membrane fusion is controlled by OPA1 and gives rise to the interconnected mitochondrial reticulum. Fission results in division of a mitochondrion into two smaller mitochondria and is required for mtDNA distribution during mitochondrial division and the removal of damaged mitochondria via mitophagy. Dynamin-related protein 1 (Drp1) is a key regulator of mitochondrial fission and is recruited from the cytoplasm to the outer mitochondrial membrane where it oligomerizes and forms a ring-like structure that constricts and severs the mitochondrion (Breitzig et al., 2018; Zerihun et al., 2023). Changes in mitochondrial shape affect cellular energy metabolism and mitochondrial division is thought to be important for partitioning of damaged mitochondria and removing them from the mitochondrial reticulum. Our earlier studies suggest that Akap1 overexpression and modulation of Drp1 phosphorylation at Ser637 is an important therapeutic strategy for ALI (Soundararajan et al., 2022).

### 5.1 Fusion and fission processes in mitochondria

Mitochondria form a highly dynamic network within the cell through continuous processes of fusion and fission. Fusion is the process through which individual mitochondria combine into a singular network, allowing for the mixing of contents and DNA throughout the network (Chan, 2012). This is achieved through the merging of the mitochondrial outer membrane and inner membrane mediated by GTPases Mfn1 and 2 (Mfn 1/2) and OPA1, respectively (Liu et al., 2020). Changes in morphology are associated with alterations in cellular conditions. Undergoing fission, the process through which mitochondria divide, forming separate mitochondria is a swift response to isolated cellular stress, forming smaller mitochondria, which may be selectively removed via autophagy. Fission is mediated by the recruitment of Drp1 from the cytosol to the outer membrane, forming a contractile ring, which cleaves the mitochondrion (Losó n et al., 2013). Fission is a process of vital importance to the cell, allowing for removal of damaged mitochondria and isolation of damaged portions of mitochondria to prevent spread of detrimental products to unaffected mitochondria (Chan, 2012).

The balance of fusion and fission is critical to the maintenance of a functional mitochondrial network. Excessive fission may lead to the production of non-functioning mitochondria, which may be removed from the network through autophagy or may merge with functional mitochondria, potentially spreading dysfunction. The loss of Mfn2 has been shown to cause an increase in unopposed Drp1 activity and subsequent increased mitochondrial fragmentation and ROS production. Mice lacking Mfn 1 and 2 in the skeletal muscle have been shown to have a dramatic reduction in mitochondria content and reduced oxygen consumption. OPA1 has a role in maintaining cristae structure and DNA integrity in addition to its role in fusion, with loss of OPA1 causing mitochondrial fragmentation and autophagy of the damaged mitochondria (Meeusen et al., 2006). While fusion is generally thought to be protective in times of stress by allowing content mixing and sharing of healthy mitochondria to counteract damage to others, it has also been shown to increase the spread of detrimental mtDNA in a model of sepsis, suggesting it may exacerbate certain inflammatory conditions.

### 5.2 Influence of altered mitochondrial dynamics on inflammation

Altered mitochondrial dynamics, which include the processes of mitochondrial fission, fusion, biogenesis, and mitophagy, play a critical role in regulating cellular homeostasis and can significantly influence inflammatory responses (Chen W. et al., 2023b). Smooth transition from M1 to M2 macrophages is important for the resolution of inflammation, which is driven by a change from glycolysis to oxidative phosphorylation and an increase in OPA1 and Mfn to promote fusion (Mills and O’Neill, 2016). Prolonged inflammation caused by lack of clearance of apoptotic cells by M2 macrophages is central to a number of diseases such as COPD and asthma (Liu et al., 2023). Mitochondrial ROS production may also serve as a signal to augment inflammatory gene expression. It is suggested that induction of iNOS in response to ROS causes peroxynitrite-mediated damage to OPA1, sequentially leading to mitochondrial fragmentation, cytochrome c release and subsequent apoptosis (Di Meo et al., 2016). In a study by Park et al. (2015), it was shown that excessive mitochondrial fission induced by Drp1 activation leads to increased production of mitochondrial ROS, which activates the NLRP3 inflammasome, promoting inflammation (Park et al., 2015). On the other hand, mitochondrial fusion, regulated by Mfn1 and Mfn2 OPA1, helps maintain mitochondrial function and integrity. Impaired fusion can result in fragmented mitochondria, mitochondrial dysfunction, and increased susceptibility to stress, leading to enhanced inflammatory responses. Yu et al. demonstrated that the loss of Mfn2 in macrophages leads to mitochondrial fragmentation and dysfunction, resulting in increased production of pro-inflammatory cytokines and an exaggerated inflammatory response (Yu et al., 2008). St-Pierre et al. found that overexpression of PGC-1α in muscle cells reduced oxidative stress and inflammation, suggesting that promoting mitochondrial biogenesis can have anti-inflammatory effects (St-Pierre et al., 2006).

Mitophagy is the selective autophagic removal of damaged mitochondria, crucial for mitochondrial quality control. Defective mitophagy can lead to the accumulation of dysfunctional mitochondria, increased mtDNA release, and heightened inflammatory responses. Sliter et al. showed that impaired mitophagy due to Parkin or PINK1 deficiency leads to the accumulation of damaged mitochondria and increased inflammation via activation of the cyclic GMP-AMP synthase - stimulator of interferon genes (Sliter et al., 2018).

Several studies have been demonstrated the specific role of mitochondrial dynamics in COPD (Cloonan and Choi, 2016) and asthma (Mabalirajan et al., 2008). There is difficulty in discerning cause and effect with altered mitochondrial dynamics and inflammation, as evidenced by conflicting results in a number of disease models using Mfn2^−/−^ mice (Sorrentino et al., 2018; Khodzhaeva et al., 2021). Overall there is a clear link between altered mitochondrial dynamics and inflammation, and mitochondrial fission or fusion may present as a novel therapeutic target for inflammatory lung diseases. Fission has the advantage of producing more mitochondria and therefore more ATP, which may be beneficial in diseases involving energy depletion of which asthma is one (Trian et al., 2007; Xu et al., 2016). With the advancement of research in this field, targeting a specific process allows avoidance of global changes influenced by previous strategies such as glucocorticoids and can neatly reverse a phenotype by re-establishing a balance between fission and fusion.

### 5.3 Therapeutic targeting of mitochondrial dynamics

Similar to how fusion can be manipulated to enhance bioenergetic function, fission can also be manipulated in a way that changes the overall function of the mitochondria. Because the process of fission is usually upregulated in a variety of disease states to propagate mitochondrial damage, preventing fission has the potential to preserve the functional mitochondria and improve cellular function. This has been proven in a study using Mdivi-1, a selective inhibitor of the fission protein Drp1, that has shown to protect against neural cell death in an animal model of Parkinson’s disease (Zhou et al., 2018). Although the process of fission is typically thought to be detrimental, fission is necessary in the removal of damaged mitochondria such that they are not a source of cellular toxins. This presents a difficult balance in regulating fission to prevent further damage while still promoting the removal of damaged mitochondria.

The process of fusion can be manipulated by upregulating OPA1 to promote retention of mtDNA and enhance bioenergetic function in cells. It can also be promoted by overexpression of MFN to enhance ATP production and reduce ROS generation in the heart. Mitochondria-targeted antioxidants have also been proven to enhance mitochondrial fusion that improves bioenergetic capacity and reduces oxidative damage. A recent study has shown that Mito-TEMPO, a scavenger of mitochondrial O^2-^, has the ability to ameliorate angiotensin II-mediated vascular endothelial dysfunction by enhancing mitochondrial fusion and OPA1 expression (Dikalova et al., 2010). Preserving the structural integrity of mitochondria has also been shown to reduce apoptosis and cell death associated with a variety of disease states.

Mitochondrial dynamics play a crucial role in the pathophysiology of various inflammatory lung diseases, including asthma, COPD, ALI (Cloonan and Choi, 2016). Targeting mitochondrial dysfunction offers potential therapeutic strategies for these conditions. In asthma, mitochondrial dysfunction is linked to increased mtROS and alterations in mitochondrial morphology, including enhanced fission and reduced fusion. Therapeutic strategies focus on reducing oxidative stress through mitochondrial antioxidants and utilizing mesenchymalstromal cells (MSCs) to transfer healthy mitochondria, which can mitigate inflammation and airway hyperresponsiveness (Cloonan and Choi, 2016; Caldeira et al., 2021).

COPD involves significant mitochondrial alterations such as reduced oxidative phosphorylation (OXPHOS) and mitochondrial fragmentation. Cigarette smoke (CS) exposure exacerbates these issues, leading to increased mtROS and cellular damage. Therapeutic approaches include promoting mitochondrial fusion through agents like leflunomide, which enhances the function of Mfn2, a key protein in mitochondrial fusion (Miret-Casals et al., 2018). Mdivi-1, a compound that inhibits mitochondrial division and mitophagy, effectively reduced cell mortality and mitochondrial dysfunction caused by CS *in vitro*. Additionally, it provided protection against mitochondrial fragmentation and lung fibrosis generated by bleomycin in mice (Mizumura et al., 2014; Carter, 2017). These findings indicate that drugs that suppress Drp1 could be beneficial for treating illnesses characterized by excessive mitochondrial fission.

In ALI, mitochondrial dysfunction contributes to impaired cell bioenergetics and heightened oxidative stress. Targeting mitochondrial pathways to enhance bioenergetic function and reduce mtROS production can help in managing ALI. Interventions may include the use of mitochondrial-targeted antioxidants and regulators of mitochondrial dynamics to restore normal function and mitigate lung injury (Sheu et al., 2006). Recent research indicates that mitochondrial Akap1 and BMI1, a protein present in the Polycomb repressive complex 1, have been identified to affect mitochondrial function. The expression of these proteins has been drastically reduced in human lung epithelial cells and mice exposed to hyperoxia conditions. The observed decrease was associated with an elevation in PINK1, Parkin, and DJ1 (protein deglycase), indicating that Akap1 and BMI1 could be promising targets for therapeutic intervention in the treatment of ALI/ARDS (Narala et al., 2018; Hernández-Cuervo et al., 2022; Soundararajan et al., 2022).

Overall, therapeutic strategies that target mitochondrial dynamics and function, such as enhancing fusion, reducing fission, and controlling oxidative stress, hold promise for treating these inflammatory lung diseases. These approaches aim to restore mitochondrial integrity and function, thereby improving cellular resilience and reducing inflammation.

## 6 Mitochondrial metabolism in inflammatory lung diseases

In health, the predominant form of metabolism in lung cells occurs via oxidation of these fuels in the mitochondria to generate ATP, which is a highly efficient process. During inflammation, however, particularly if cells are activated to divide and synthesize new proteins, there is a switch to higher rates of glycolysis in order to generate metabolic intermediates for these processes. Higher rates of aerobic glycolysis in activated immune cells are also thought to be an important pro-inflammatory effector mechanism (Soto-Heredero et al., 2020). This phenomenon was first described by Otto Warburg in cancer cells and is now being recognized as a ubiquitous feature of immune cell activation (Warburg, 1956).

Mitochondria are unique in that they possess their own DNA and protein synthesis machinery, but they are also intimately involved in the control of the nucleus. As a consequence, dysfunctional mitochondria can initiate cell death or the increased production of pro-inflammatory mediators that can have profound effects on neighboring cells. The function of mitochondria is largely determined by the metabolic processes that occur within them. These can be broadly divided into catabolic processes that occur in the cytoplasm in order to generate fuels in the form of glucose or fatty acids, and the anabolic process of synthesizing ATP and other high energy phosphate bonds.

Mitochondria are the main power source of the cell and are increasingly being recognized as key players in inflammation. By regulating cellular energy metabolism, they control the production of many pro-inflammatory mediators.

### 6.1 Warburg effect and inflammation

The shift from normal mitochondrial function to the Warburg effect has been observed in several immune cells in pulmonary inflammation. Rodriguez-Prados et al. used a microarray analysis of thioglycollate-induced peritoneal macrophages and LPS-induced alveolar macrophages to compare their gene expression with *in vivo* inflammatory macrophages (Rodríguez-Prados et al., 2010). They found a common transcriptional profile characteristic of a decrease in mitochondrial oxidative metabolism and an increase in glycolysis, showing that although *in vitro* and *in vivo* macrophages encountered different environmental conditions, they both utilized the Warburg effect to provide a suitable inflammatory response. A similar study showed that in asthmatics, bronchoalveolar lavage (BAL) T lymphocytes have decreased mitochondrial membrane potential and depolarization of their mitochondria, indicating a metabolic switch to glycolysis (Zhou et al., 2021).

During inflammation, immune cells require increased glucose metabolism to provide the resources necessary for rapid proliferation and cytokine production. Although glycolysis is an inefficient pathway for glucose metabolism compared to oxidative phosphorylation, it can be sustained at a higher rate to provide ATP when the rate of glucose uptake is increased. This is essential for immune cells which may encounter regions of hypoxia in tissue microenvironments. ATP generated from increased glycolysis can be used without oxygen and is independent of mitochondrial ATP production. In this context, the Warburg effect actually bypasses the need for mitochondrial function, thus immune cells can inhibit normal mitochondrial activity and switch off respiration to simply use glycolysis as an energy source. This is illustrated in a study showing that in LPS-induced macrophages, although the rate of glucose consumed was the same as resting macrophages, the rate of lactate produced was much higher, indicating increased glycolysis while suppressing mitochondrial glucose metabolism (O’Neill and Hardie, 2013).

In recent years, the Warburg effect has been linked to inflammatory phenotypes in autoimmune diseases and chronic infectious diseases (including asthma and COPD) as well as cancer, showing that aberrant immune responses and the property of immune cells to divide and proliferate can utilize the same metabolic pathways to provide fuel for increased effector responses (Figure 1).

### 6.2 Potential therapeutic strategies targeting metabolic reprogramming

Several potential strategies targeting metabolic pathways in lung immune/inflammatory response are conceivable. As immune and inflammatory responses are highly energy consuming, it is important to modulate these with respect to the metabolic state of the cell. Provided that the immune responses are complex and vary dependent on environmental cues, it is unlikely that systemic suppression of immune response will be beneficial for patients with inflammatory lung diseases. However, in diseases where chronic immune activation causes tissue damage, strategies aimed to inhibit immune/inflammatory cell recruitment, rather than function, may be useful. The mammalian target of rapamycin (mTOR) is an important regulator of cell metabolism and is involved in immune cell differentiation and function. Inhibition of mTOR with agents such as rapamycin would be expected to switch immune cell metabolism from glycolysis to oxidative phosphorylation and therefore may be detrimental to effector T cells but beneficial to regulatory T cells and macrophages in a Th1 or M1 state (Panwar et al., 2023). Rapamycin has been shown to attenuate lung inflammation in several animal models and in a small clinical trial of patients with hypersensitivity pneumonitis, it reduced lymphocytic BAL count and improved lung function (Paddenberg et al., 2007; Yan et al., 2016). An alternative approach to inhibiting immune cell recruitment may be to target glycolysis directly. PFK-15 is an inhibitor of PFKFB3, a key regulator of glycolysis which produces F2,6P, an allosteric activator of glycolysis. It has been shown to be effective in murine models of rheumatoid arthritis and may be beneficial in other chronic inflammatory diseases. Similarly, inhibition of lactate dehydrogenase has proved effective in reducing inflammation in a murine model of colitis. These strategies are currently of interest to the pharmaceutical industry and may be applicable to inflammatory lung diseases. An exciting recent development has been the discovery of itaconate as an anti-inflammatory metabolite which inhibits the succinate-hypoxia-inducible factor-1α axis (Lampropoulou et al., 2016). This discovery may open possibilities for novel therapeutic approaches in the future.

## 7 Mitochondrial quality control and inflammation

Mitochondrial quality control provides essential homeostatic functions including the regulation of the mitochondrial population to meet cellular energy demands, as well as the disposal of damaged mitochondria (Marchi et al., 2023). There are several cellular systems in place to ensure the maintenance of a healthy population of functional mitochondria. When damage occurs, it is often possible for the mitochondria to undergo membrane permeability transition and release proapoptotic proteins. In this event, it is considered more favourable for the mitochondria to be degraded and removed, rather than for the cell to undergo apoptosis. In this instance, the removal of the mitochondria involves disassembly of the organelle back into its constitutive proteins, followed by degradation in the cytosol, therefore bypassing the apoptotic pathway. This process is known as mitophagy, a selective form of autophagy for the removal of mitochondria. Global inhibition of apoptosis in the epithelium can reduce airway inflammation and injury without concomitant increase in cell proliferation (White, 2011). Apoptotic epithelial cells can serve as a source of injury to the lung either via direct cytotoxic effects on neighbouring cells, or by the release of proinflammatory intracellular molecules. Therefore, in circumstances where the mitochondria have triggered an apoptotic cascade, removal of the organelle to prevent further injury to the lung may be a more favourable outcome. Finally, should the mitochondria sustain minor damage, there are repair mechanisms in place to ensure the mitochondrial population remains functional and undiminished.

### 7.1 Role of autophagy in mitochondrial quality control

Mitochondria are the major source of energy in the form of adenosine triphosphate to fuel cellular processes. In addition, they are involved in many other biological processes and have been described as the powerhouses of the cell. In order to perform these roles effectively, mitochondria need to be in good condition. However, mitochondria can be damaged by a variety of insults, including oxidative stress, and damaged mitochondria may release harmful substances, amplifying the injury to the cell. On the other hand, pathway regulation, autophagy elimination of unhealthy mitochondria, and biogenesis of new mitochondria ensure mitochondrial protection. Thus, the events and regulating smooth transition in between can greatly affect the severity of lung diseases. Autophagy is an evolutionarily conserved process for degradation and recycling of cellular components in response to various forms of stress and known to take place in damage control for mitochondria. Recently, it has been clarified that autophagy can eliminate injured mitochondria while inducing biogenesis of new mitochondria, showing that the control of mitochondria number is not solely dependent on biogenesis, also giving a new interpretation for mitochondria control (Li A. et al., 2022b).

### 7.2 Mitophagy and inflammation

Mitophagy is the selective degradation of mitochondria by autophagosome, which prevents an excessive inflammatory response. The elimination of damaged mitochondria prevents the release of mtDNA, which induces inflammation. Normally, PTEN-induced putative kinase 1 (PINK1) is continuously degraded, and thus kept at a low level. When damage to mitochondria occurs, PINK1 accumulates on the mitochondrial membrane and initiates the process of mitophagy (Jin and Youle, 2012). A variety of receptors mediate the removal of damaged mitochondria. For example, Bnip3 and Nix induce mitophagy by activating macroautophagy through the facilitation of LC3 on the mitochondrial membrane (Field and Gordon, 2022). Parkin is an E3 ubiquitin ligase which attaches ubiquitin to damaged mitochondria to mark them for removal. FUNDC1, a receptor which is attached to the mitochondria, is activated by hypoxia, and induces mitophagy.

### 7.3 Impaired mitochondrial quality control in lung diseases

In an attempt to protect mitochondrial function from further damage, cells have signaling pathways to induce the arrest of an organelle and prevent its division. A damaged organelle that has surpassed its divisional capacity is more likely to be removed (Ng et al., 2021). The division and replication of the mitochondrial pool are pivotal to cellular energy demands and often dictate cell fate. If a damaged mitochondrion is dividing at the time of a harmful insult, there is a chance that both daughter mitochondria will also be damaged. In damaged mitochondria, reduction of the organelle membrane potential is a likely signal for arrest and prevention of division. Mitochondria possess several complex quality control systems to maintain their functional integrity (Yamano et al., 2024). When these attempts fail, the organelle will be targeted for removal by autophagy, a process known as mitophagy.

The impairment of Mitochondrial Quality Control and resulting mitochondrial dysfunction are strongly linked to age-related diseases and respiratory disorders, including asthma, COPD, idiopathic pulmonary fibrosis and lung cancer (Sharma et al., 2021; Liu et al., 2023). Mitochondrial quality control includes a series of processes involving the maintenance of mitochondrial function and removal of damaged mitochondria. Under cellular stress conditions, damaged mitochondria continuously produce ROS which may further damage mtDNA, proteins, and lipids (Yakes and Van Houten, 1997). The ability to remove damaged cellular constituents is vital to cell survival. Cells have developed intricate systems to sense and remove damaged mitochondria (Narendra et al., 2008). When damage is episodic and mild, the capacity of the organelle to repair itself is often sufficient to maintain homeostasis. When damage is severe or chronic, removal of the organelle in question is often the best solution. If the damaged mitochondria cannot be removed, further detrimental effects may occur and often contribute to the progression of inflammatory lung diseases. Recent research findings on mitochondrial quality control in lung diseases have been reviewed elsewhere (Liu et al., 2023; Li et al., 2024; Liu et al., 2024). Maintenance of a functional mitochondrial pool is therefore an important defense against cellular injury. Some of the mechanisms are represented in Figure 1.

## 8 Therapeutic approaches targeting mitochondria in inflammatory lung diseases

Pharmacological interventions have been the most widely studied potential therapeutic approach to target mitochondria in inflammatory lung diseases (Li et al., 2013; Liu and Chen, 2017). They are generally directed against specific components of the mitochondrial signal transduction pathway, and these agents might not be specific to lung disease and might also have off-target effects. A prime example would be the use of N-acetyl cysteine (NAC) as a mucolytic agent in chronic airway diseases (Silveira et al., 2019; Mokra et al., 2023). The antioxidant activity of NAC is directly attributed to the presence of the thiol (-SH) group and acetyl group attached to the amino group (Rushworth and Megson, 2014). These chemical groups enable NAC to effectively interact with a wide range of radical and non-radical reactive oxygen and nitrogen species (RONS).

NAC has been shown *in vitro* to prevent mitochondrial permeability transition and cytochrome c release in response to apoptosis-inducing stimuli in a lung epithelial cell line, In a recent *in vitro* investigation, cigarette smoke-induced CFTR failure hindered alveolar macrophages’ ability to phagocytose (Ni et al., 2015). CS also reduced mitochondrial respiration while promoting glycolysis and the production of ROS. NAC, however, diminished these effects (Aridgides et al., 2019) and *in vivo* administration in a smoke-exposed rat model of emphysema prevented increases in lung iNOS expression, 3-nitrotyrosine formation, NF-κB activation, and alveolar destruction (Rushworth and Megson, 2014). Despite these promising findings, a clinical trial using NAC treatment failed to demonstrate any effect in preventing lung function decline in smokers over a 3-year period (Decramer et al., 2005). Another potential pharmacological approach has been the use of cyclosporine A, a well-established inhibitor of the MPTP, in the context of ALI in a rat model (Lin et al., 2018). Cyclosporine A-treated rats had attenuated lung edema, lung inflammation, upregulation of mitophagy related genes and improved oxygenation in comparison to untreated rats following mechanical ventilation-induced lung injury (Lin et al., 2018). Although there are no specific data on effects in mitochondria and the lung in this study, these findings could indicate a potential benefit of cyclosporine A on the lung’s response to injury through inhibition of the MPTP. More recently, interest has focused on directly targeting mitochondria with antioxidant compounds. This has been explored in a study examining the effects of the mitochondria-targeted ubiquinone derivative MitoQ on CS-induced endothelial dysfunction (Chen et al., 2019). MitoQ is formed by covalently linking ubiquinone to the lipophilic triphenylphosphonium cation, which facilitates its accumulation within mitochondria. When administered to rats, MitoQ was able to normalize mitochondrial superoxide levels, which in turn prevented the activation of oxidant-sensitive signaling pathways and attenuated the associated endothelial dysfunction (Wani et al., 2011). At present, the specific effects of MitoQ and similar compounds on the mitochondria in lung diseases still require investigation.

Multiple studies have investigated the role of ROS in ALI. As a result, several trials have focused on targeting ROS as a potential treatment for ALI and ARDS (Rice et al., 2011; Li et al., 2015). Regrettably, the presence of molecular instability is a significant obstacle in targeting ROS for therapeutic purposes. Additionally, it is important to note that attempts to reverse mitochondrial damage using antioxidant treatment, which specifically targets ROS, have proven to be ineffective (Gomes et al., 2014; Galam et al., 2015). Recent research conducted on mice has demonstrated that focusing on the proteins responsible for mitochondrial activity provides more effective protection compared to antioxidant treatment (Adlam et al., 2005; Chacko et al., 2010; Islam et al., 2012). Mitochondrial dysfunction in COPD leads to increased ROS production, contributing to oxidative stress and inflammation. Mitochondria-targeted antioxidants (MTAs) have shown potential in reducing ROS levels and protecting against mitochondrial damage. These antioxidants can mitigate inflammation and slow disease progression, which traditional antioxidants have not effectively accomplished (Fairley et al., 2023). A recent review by Chellappan et al. has elaborately discussed on mitochondrial dysfunction in chronic lung disease and highlighted potential therapeutic targets and treatment strategies (Chellappan et al., 2022). The research suggests that targeting mitochondrial function and processes could be a viable approach to develop novel treatments for various respiratory conditions affecting the lungs.

### 8.1 Pharmacological interventions targeting mitochondria

Mitochondrial dysfunction plays a significant role in various inflammatory lung diseases, including asthma, COPD, and pulmonary fibrosis. Recent research has focused on pharmacological interventions targeting mitochondrial function to mitigate these conditions (Singh et al., 2021).

#### 8.1.1 Antioxidants and mitochondrial protectants

MitoQ and MitoE are potent antioxidants specifically designed to accumulate within the mitochondria. They help reduce oxidative stress by neutralizing ROS, which are often elevated in inflammatory lung diseases. Preclinical studies indicate that these compounds can restore mitochondrial function and improve cellular health in models of asthma, IPF and COPD (Chen et al., 2019; Fairley et al., 2023; Huang et al., 2023; Li et al., 2023). Cinnamate Compounds have been shown to enhance mitochondrial function by reducing ROS production and preventing mitochondrial fission, making them potential candidates for asthma treatment (Kumar et al., 2013). Due to the ability of mitoquinone to prevent oxidative damage to mitochondrial proteins and mtDNA in addition to its ROS scavenging capabilities, it may remain a potentially useful agent in prevention of lung mitochondrial damage in the future.

#### 8.1.2 Modulators of mitochondrial dynamics

Mdivi-1 inhibits Drp1, a protein involved in mitochondrial fission. By promoting mitochondrial fusion and reducing fragmentation, Mdivi-1 has demonstrated protective effects against cell death and mitochondrial dysfunction in models of ALI and pulmonary fibrosis (Luo et al., 2019; Deng et al., 2020; Tong et al., 2024). Another selective inhibitor of Drp1, P110 has shown neuroprotective effects and improved mitochondrial integrity, suggesting its potential utility in treating diseases characterized by excessive mitochondrial fission (Qi et al., 2013).

#### 8.1.3 Metformin and other metabolic modulators

Metformin, primarily known as an anti-diabetic medication, has been found to inhibit mitochondrial ATP and DNA synthesis, which can abrogate NLRP3 inflammasome activation and reduce pulmonary inflammation. This suggests a dual role in managing metabolic and inflammatory aspects of lung diseases (Sato et al., 2016; Xian et al., 2021). L-Arginine has been shown to restore mitochondrial function in bronchial epithelial cells, improving ATP levels and mitochondrial activity in asthma models (Xu et al., 2016). Its administration may counteract the effects of peroxynitrite accumulation associated with asthma (Maarsingh et al., 2009).

#### 8.1.4 Mitochondrial transplantation

Emerging research is exploring the feasibility of mitochondrial transplantation as a therapeutic strategy to repair damaged lung tissues (Moskowitzova et al., 2020). This approach aims to restore normal mitochondrial function in diseased cells, although it remains largely experimental (Cloer et al., 2023). Pharmacological agents targeting mitochondrial function and dynamics offer new avenues for treatment, potentially improving outcomes for patients with conditions like asthma, COPD, and pulmonary fibrosis. Continued research is essential to fully elucidate the mechanisms involved and to develop effective mitochondrial-targeted therapies.

### 8.2 Nutritional strategies

Nutritional strategies aimed at improving mitochondrial function are increasingly recognized as potential complementary therapies for managing inflammatory lung diseases. Currently, the most established approaches focus on dietary antioxidants, which have been shown to neutralize ROS in various inflammatory conditions. This action helps prevent oxidative damage to mtDNA and mitochondrial proteins (Bjørklund and Chirumbolo, 2017). While there is a wealth of preclinical research addressing mitochondrial dysfunction with nutritional supplements, clinical studies involving human subjects remain limited, though they show promising potential (Zong et al., 2024).

#### 8.2.1 Antioxidants

Mitochondria-targeted antioxidants, such as MitoQ and SkQ1, have demonstrated efficacy in reducing oxidative stress and inflammation in COPD models (Chen et al., 2019; Wysoczanski et al., 2022). These compounds specifically target the mitochondria, thereby enhancing their protective effects against ROS generated during inflammation (Fairley et al., 2023; Li et al., 2024).

#### 8.2.2 Fatty acids

These polyunsaturated Omega-3 fatty acids have anti-inflammatory properties and have been shown to improve mitochondrial function. They can modulate mitochondrial dynamics and enhance bioenergetics, potentially alleviating inflammation in lung diseases (Casanova et al., 2023). Nitrated fatty acids, particularly 10-nitrooleate, have emerged as promising therapeutic agents in the context of ALI, particularly in models of hyperoxia-induced lung damage. Research indicates that 10-nitrooleate exerts protective effects by mitigating oxidative stress and inflammation, which are critical factors in the pathophysiology of ALI (Panati et al., 2020; Narala et al., 2022). This compound operates through several mechanisms, including the modulation of mitochondrial function, where it influences redox reactions and reduces ROS production. These findings highlight the potential of nitrated fatty acids as a novel therapeutic strategy for managing lung diseases, emphasizing their role in both mitochondrial protection and the reduction of inflammatory responses.

#### 8.2.3 Nutrients supporting mitochondrial biogenesis

Compounds such as coenzyme Q10 (CoQ10), alpha-lipoic acid, and PGC-1α activators have been shown to promote mitochondrial biogenesis and improve mitochondrial function through various mechanisms. These nutrients can help restore the mitochondrial network’s integrity, which is often compromised in inflammatory lung diseases.

CoQ10 is a vital component of the mitochondrial respiratory chain and acts as a potent antioxidant. It has been found to enhance mitochondrial biogenesis and improve mitochondrial function, particularly in the context of oxidative stress and cellular energy production (Hidalgo-Gutiérrez et al., 2021). Research has demonstrated that CoQ10 supplementation can increase the expression of genes involved in mitochondrial biogenesis, such as PGC-1α, nuclear respiratory factor 1, and mitochondrial transcription factor A, leading to improved mitochondrial content and function (Salehpour et al., 2019). Alpha-lipoic acid, another antioxidant, has been shown to upregulate the SIRT1-dependent PGC-1α pathway, which is crucial for mitochondrial biogenesis and function. PGC-1α activators directly stimulate the transcriptional coactivator PGC-1α, which regulates genes involved in energy metabolism and mitochondrial biogenesis. Activation of PGC-1α leads to an increase in mitochondrial content and function. These pathways enhance the expression of key mitochondrial genes and protects against oxidative stress (Salehpour et al., 2019). Vitamins such as vitamin D and vitamin E play crucial roles in mitochondrial function and may help mitigate oxidative stress. Deficiencies in these vitamins have been linked to increased severity of lung diseases, suggesting that adequate intake could be beneficial (Yavari et al., 2022).

Combining dietary interventions with lifestyle modifications, such as regular physical activity and smoking cessation can enhance mitochondrial function and improve overall lung health. Exercise has been shown to stimulate mitochondrial biogenesis and improve oxidative capacity, which may counteract the effects of chronic inflammation in the lungs (Fairley et al., 2023). Moreover, personalized nutrition strategies that consider individual genetic and metabolic profiles may optimize mitochondrial health and enhance therapeutic outcomes in patients with inflammatory lung diseases.

## 9 Bibliometric analysis

In order to examine the connection between mitochondrial dysfunction and lung disorders, we performed a comprehensive search of the Scopus database using the search term “mitochondrial dysfunction and lung.” We limited the search to publications published since 2019 to prioritize current research. This initial search returned 1,531 results.

**FIGURE 3:**
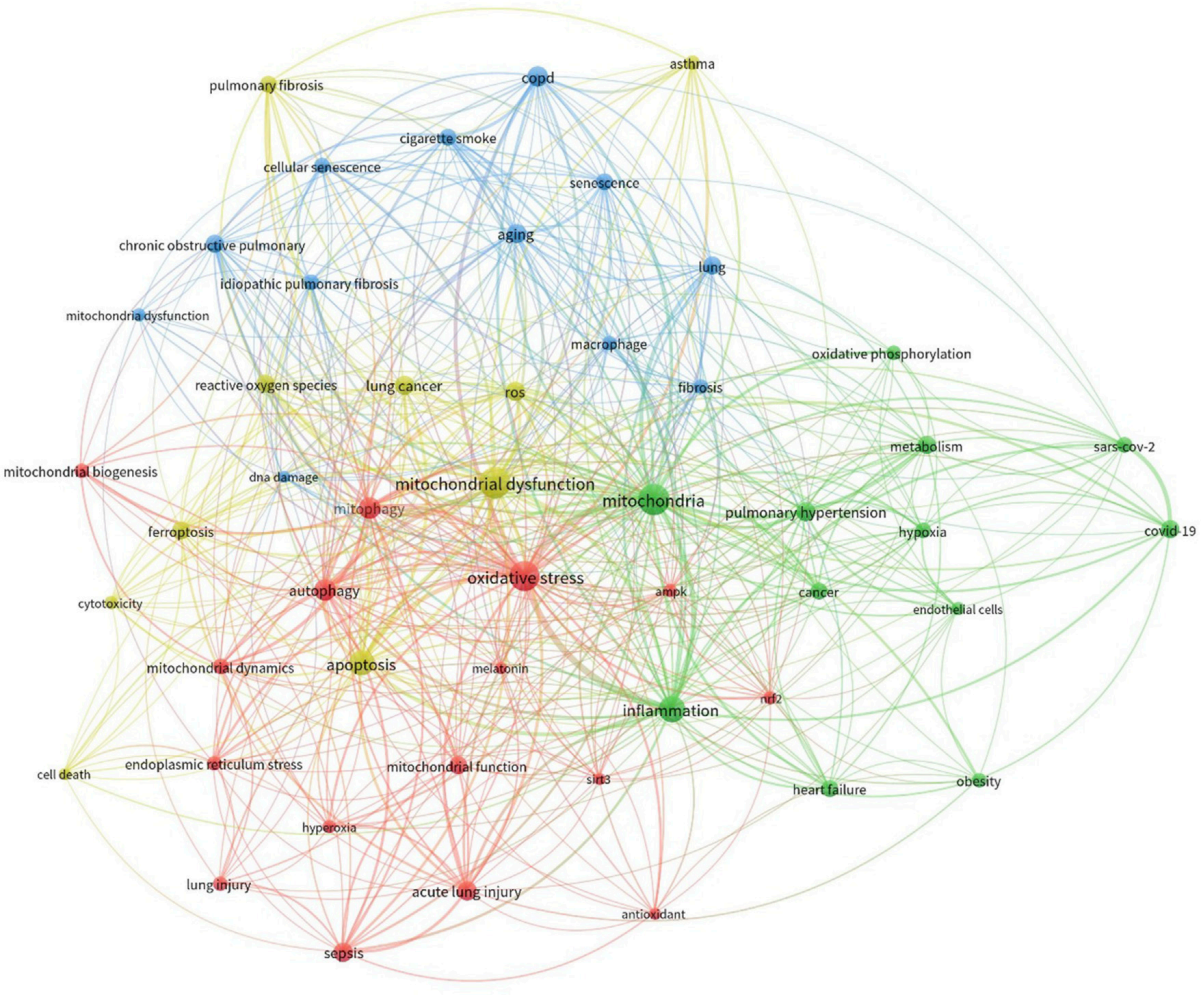
Co-occurrence analysis of keywords extracted from publications sourced from the Scopus database. The literature underwent collection and processing as detailed in the main text. The figure illustrates the hierarchical arrangement of keywords, with distinct colors representing keyword families. Larger circle sizes denote higher frequencies of occurrence.

The results were subsequently imported into R software and the Bibliometrix tool was used for bibliometric analysis (Aria and Cuccurullo, 2017). The analysis centered on keyword co-occurrence, which assessed the frequency with which keywords appeared together within the retrieved literature. A keyword co-occurrence network was then visualized using VOSviewer software (Figure 3). To enhance clarity, the analysis included only the top 50 most frequently occurring keywords in the network visualization. Additionally, the keywords were normalized using association strength and clustered them for further analysis (resolution: 1).

We have determined the significant disease conditions and molecules linked to both mitochondrial dysfunction and lung disorders through an analysis of the frequency of appearance and importance within the network of co-occurring elements. The analysis identified the emergence of four distinct clusters. The data clearly showed a significant correlation between mitochondrial dysfunction and mitochondrial dynamics, including mitophagy and biogenesis. Moreover, the network exhibited the emergence of processes such as ROS generation, apoptosis, and DNA damage. Ultimately, we also discovered a group consisting of other lung diseases like COPD, ALI, asthma, and fibrosis. This bibliometric research, which employed keyword co-occurrence, offered valuable insights into the possible correlation between mitochondrial dysfunction and the onset of lung diseases.

## 10 Future directions for research

While basic science research findings have been achieved, not much translational research has been reported based on these findings. Examples could include the development of sophisticated animal models that mimic human conditions and studies using mitochondria-targeted antioxidants or drugs aimed at specific mitochondrial proteins. Studies such as these will provide a clearer understanding of the importance of these findings on lung disease as a whole, and these will provide the insights needed to convince pharmaceutical companies to sponsor drug trials aimed at specific mitochondrial defects in lung disease. Other important areas to be focused on are identifying and validating biomarkers for diagnosis and treatment monitoring and exploring novel therapeutic targets such as antioxidants and mitochondrial biogenesis stimulators. Additionally, studies should address genetic and epigenetic influences on mitochondrial function and the interplay between mitochondrial dysfunction. Finally, understanding mitochondrial dynamics and the crosstalk between lung mitochondria and other organs will provide insights into the systemic effects of mitochondrial dysfunction in lung diseases.

## 11 Conclusion

Overall, mitochondria are active participants in the development of inflammatory responses in lung diseases. They contribute to the release of pro-inflammatory mediators, cell activation, and phagocytosis, and are involved in sensing and amplifying the inflammatory signal. There is evidence that mitochondrial ROS concentrate locally and lead to activation of the caspase-independent cell death program, apoptosis inducing factor-mediated cell death, and/or necrotic cell death. The death of structural or immune cells can lead to a pro-inflammatory response, either as a sequela to activation of the resolution phase of inflammation or due to post-injury exposure of the lung to injurious stimuli. This may lead to perpetuation of the inflammatory response and ongoing mitochondrial damage. Furthermore, the majority of research examining the characteristics of treatments that impact mitochondria is at the preclinical stage. Therefore, it is necessary to conduct additional extensive studies to examine the benefits of potential therapies that impact mitochondria, in order to definitively determine their efficacy.

## Figures and Tables

**Table:** 

**ACO-2**	Aconitase-2
**Akap1**	A-kinase anchoring protein1
**ALI**	Acute lung injury
**ARDS**	Acute respiratory distress syndrome
**COPD**	Chronic obstructive pulmonary disease
**CS**	Cigarette smoke
**DAMP**	Damage-associated molecular pattern molecule
**Drp1**	Dynamin-related protein 1
**ETC**	Electron transport chain
**ICS**	Inhaled corticosteroids
**IL**	Interleukin
**iNOS**	Inducible nitric oxide synthase
**IPF**	Idiopathic pulmonary fibrosis
**LABA**	Long-acting beta-agonist
**LPS**	Lipopolysaccharide
**MFN2**	Mitofusin-2
**MnSOD**	Manganese superoxide dismutase
**MPTP**	Mitochondrial permeability transition pore
**MSC**	Mesenchymalstromal cells
**MTA**	Mitochondria-targeted antioxidants
**mtDNA**	Mitochondrial DNA
**mTOR**	Mammalian target of rapamycin
**MyD88**	Myeloid differentiation primary response 88
**NAC**	N-acetyl cysteine
**NADPH**	Nicotinamide adenine dinucleotide phosphate
**NF-κB**	Nuclear factor kappa B
**NLRP3**	NLR family pyrin domain containing 3
**OMM**	Outer mitochondrial membrane
**OXPHOS**	Oxidative phosphorylation
**PGC-1α**	Peroxisome proliferator-activated receptor gamma coactivator 1-alpha
**PINK1**	PTEN-induced putative kinase 1
**RONS**	Reactive oxygen and nitrogen species
**ROS**	Reactive oxygen species
**TGF-β**	Transforming growth factor beta
**TLR9**	Toll-like receptor 9
**TNF-α**	Tumor necrosis factor alpha
